# Control of the galactose-to-glucose consumption ratio in co-fermentation using engineered *Escherichia coli* strains

**DOI:** 10.1038/s41598-020-69143-3

**Published:** 2020-07-22

**Authors:** Hyeon Jeong Seong, Ji Eun Woo, Yu-Sin Jang

**Affiliations:** Division of Applied Life Science (BK21 Plus Program), Department of Agricultural Chemistry and Food Science Technology, Institute of Agriculture & Life Science (IALS), Gyeongsang National University, Jinju, 52828 Republic of Korea

**Keywords:** Industrial microbiology, Metabolic engineering

## Abstract

Marine biomasses capable of fixing carbon dioxide have attracted attention as an alternative to fossil resources for fuel and chemical production. Although a simple co-fermentation of fermentable sugars, such as glucose and galactose, has been reported from marine biomass, no previous report has discussed the fine-control of the galactose-to-glucose consumption ratio in this context. Here, we sought to finely control the galactose-to-glucose consumption ratio in the co-fermentation of these sugars using engineered *Escherichia coli* strains. Toward this end, we constructed *E. coli* strains GR2, GR2P, and GR2PZ by knocking out *galRS, galRS-pfkA,* and *galRS-pfkA-zwf*, respectively, in parent strain W3110. We found that strains W3110, GR2, GR2P, and GR2PZ achieved 0.03, 0.09, 0.12, and 0.17 galactose-to-glucose consumption ratio (specific galactose consumption rate per specific glucose consumption rate), respectively, during co-fermentation. The ratio was further extended to 0.67 by integration of a brief process optimization for initial sugar ratio using GR2P strain. The strategy reported in this study will be helpful to expand our knowledge on the galactose utilization under glucose conditions.

## Introduction

Excessive use of fossil fuel has caused global warming, which is one of the biggest problems currently facing humanity. Recently, biobased fuels and chemicals have gained attention as potential alternatives to fossil fuels^1–6^. Some biobased fuels and chemicals can be produced via microbial fermentation from sustainable biomasses, such as wood and seaweed^7–11^.

In addition to such cellulosic biomasses, marine biomasses have been suggested as promising renewable resources^12^, as they have the advantages of high biomass yield per unit area, high rates of carbon dioxide fixation, and natural abundance^13^. Marine macroalgae contain fermentable sugars, such as glucose and galactose whose contents are known about 20% and 23%, respectively^14–18^. The proper utilization of galactose is important for the production of biobased fuels and chemicals from a marine macroalgal biomass. Also, given that galactose is 10 times more expensive than glucose, proper regulation of the galactose consumption rate is very important in the co-fermentation of glucose and galactose. On the other hand, in our recent study, it is reported that the production of some metabolite, such as hyaluronic acid could also be affected by the modulation of galactose and glucose consumption^19^. However, the exact determination of galactose and glucose consumption rates remains, in cultures using the metabolically engineered cells, yet.

Most microorganisms use glucose as a primary feedstock in the co-fermentation of glucose and galactose. This preferential consumption of glucose, which occurs through carbon catabolite repression (CCR), makes it difficult for organisms to use glucose and galactose simultaneously. In a recent study, the CCR pathway was found to be completely deregulated when glucose and galactose were co-fermented using an engineered *Escherichia coli* with knockout of the galactose repressor (*galR*) and overexpression of the galactose operon (*galP*, *galE*, *galT*, *galK* and *galM*) and phosphoglucomutase (*pgm*)^20^. The engineered strain simultaneously assimilated galactose and glucose in the co-fermentation of these sugars. However, no previous report has described a strategy for finely controlling the galactose consumption rate during the co-fermentation of glucose and galactose.

In this study, as a proof-of-concept, we tried to finely control the galactose-to-glucose consumption ratio during the co-fermentation of both sugars by engineering the sugar metabolism in *E. coli* on the basis of our recent study^19^, followed by a brief process optimization for sugar ratio and concentration.

## Results

### Galactose-to-glucose consumption ratio in *E. coli* GR2, which lacked the galactose operon repressors, during glucose consumption

**Figure 1:**
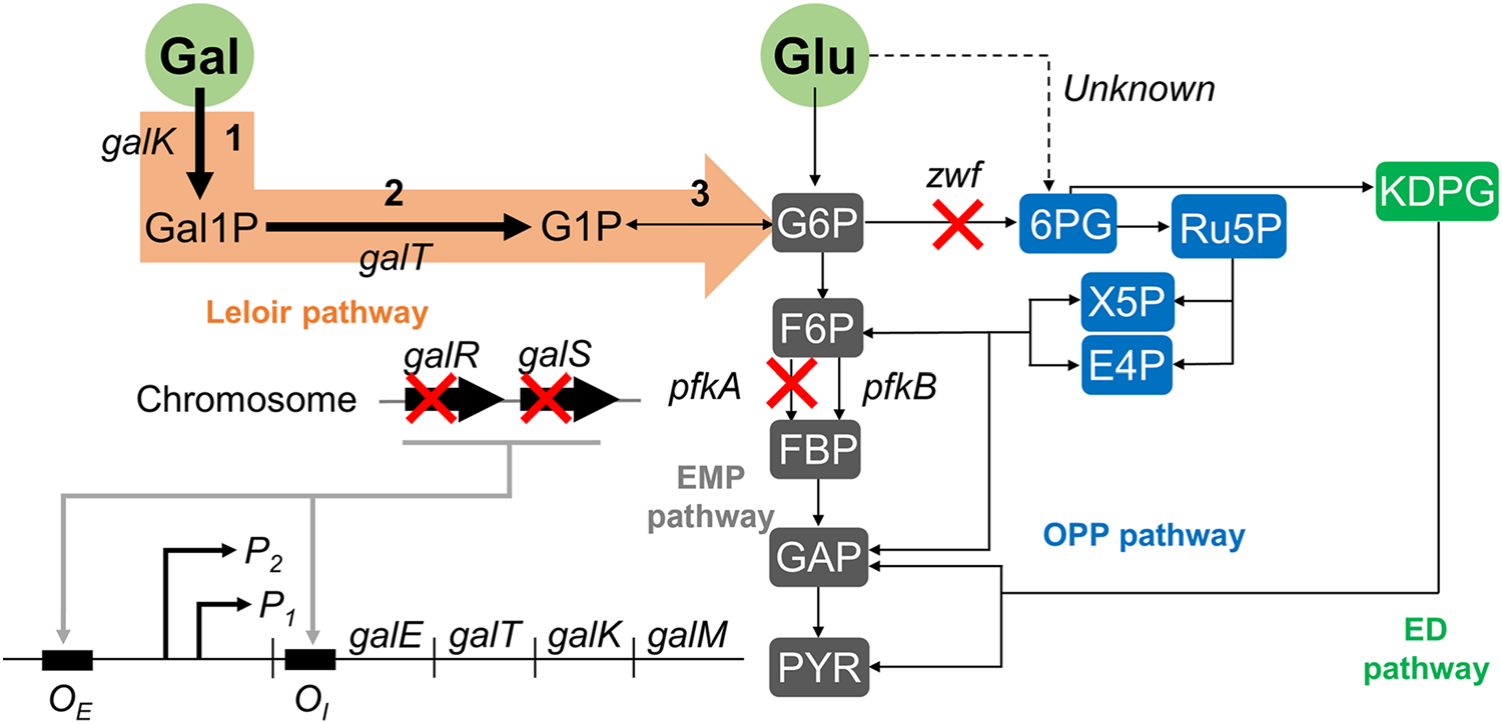
Control of the galactose-to-glucose consumption ratio in the co-fermentation of both sugars using the engineered *E. coli*. ‘X’ indicates gene knockout. The relevant genes and their encoded enzymes are as follows: *galR*, DNA-binding transcriptional repressor; *galS*, DNA-binding transcriptional isorepressor; *galK*, galactokinase; *galT*, galactose-1-phosphate uridylyltransferase; *pfkA*, 6-phosphofructokinase I; *pfkB*, 6-phosphofructokinase II; *zwf*, glucose-6-phosphate dehydrogenase. Abbreviations: Gal1P, galactose-1-phosphate; G1P, glucose-1-phosphate; G6P, glucose-6-phosphate; F6P, fructose-6-phosphate; FBP, fructose-1,6-phosphate; PYR, pyruvate; 6PG, 6-phosphate-gluconate; Ru5P, ribulose-5-phosphate; X5P, xylulose-5-phosphate; E4P, erythorse-4-phosphate; KDPG, 2-keto-3-deoxy-phosphogluconate.

**Figure 2:**
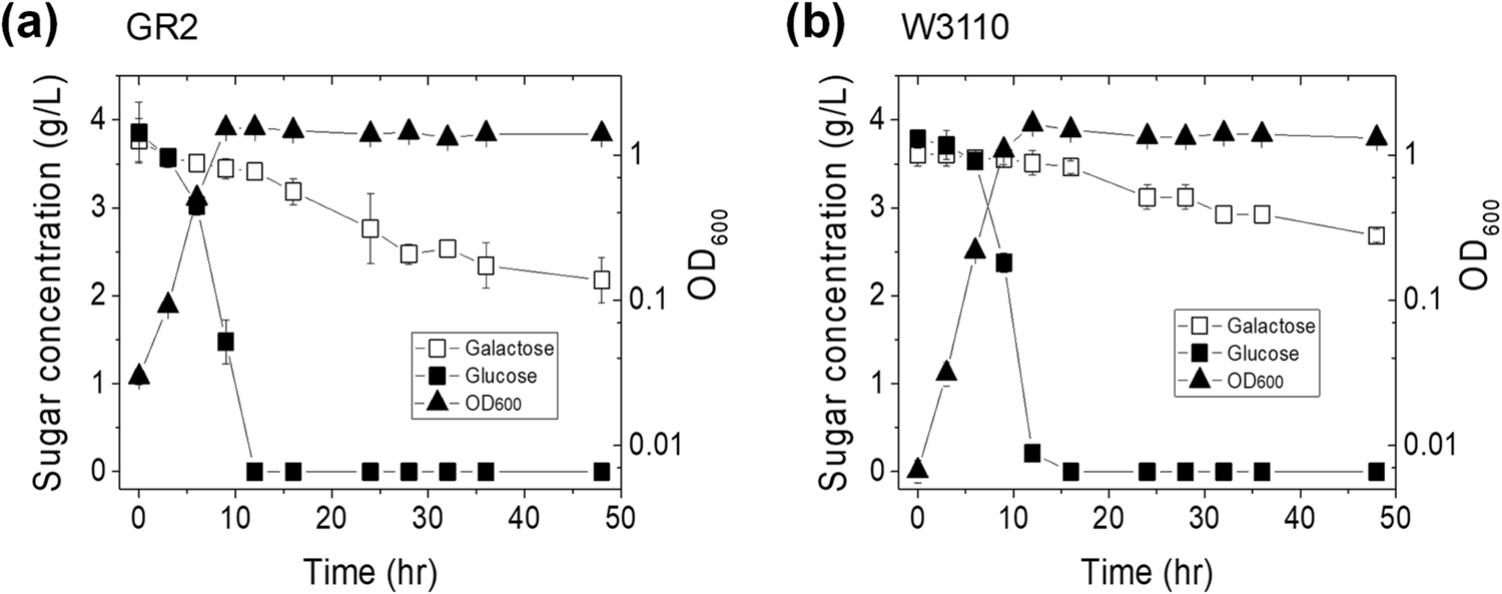
Time profiles of various parameters tested for *E. coli* strains (**a**) GR2 and (**b**) W3110 grown in R/2 medium containing 4 g/L glucose and 4 g/L galactose.

In an effort to finely control the galactose consumption rate in the co-fermentation of glucose and galactose using *E. coli*, we first constructed the *galRS* mutant, *E. coli* strain GR2 (Fig. 1 and Table 1), which was not able to produce the galactose operon repressors encoded from the *galRS* genes^21–27^. We expected that the resulting strain would consume galactose at a higher rate than the parent strain in the co-fermentation of glucose and galactose, although we anticipated that this rate would still be fairly low. To determine the galactose consumption rate of the *galRS* mutant, we cultured GR2 cells in R/2 medium supplemented with 4 g/L glucose and 4 g/L galactose. Parent strain W3110 was cultured under the same conditions as a control. In the co-fermentation of glucose and galactose, *E. coli* strain GR2 had completely consumed the glucose at 12 h of culture, yielding a specific glucose consumption rate of 1.3629 g/gDCW/h (Fig. 2a and Table 2). During this period, GR2 consumed 0.36 g/L galactose, for a specific consumption rate of 0.1262 g/gDCW/h. Under the same conditions, parent strain W3110 showed a similar level of glucose consumption (Fig. 2b) but had a lower specific galactose consumption rate at 0.0373 g/gDCW/h (Table 2). Strain GR2 yielded R_gal/glu_ = 0.09, whereas parent strain W3110 yielded R_gal/glu_ = 0.03 (Table 2).

**Table 1:** *Escherichia coli* strains and plasmids used in this study.

Name	Genotype	Reference
***E. coli*** **strains**		
W3110	Coli genetic stock center strain no.4474	CGSC^a^
GR	*E. coli* K12 W3110, △*galR*	This study
GR2	*E. coli* K12 W3110, △*galR,* △*galS*	This study
GR2P	*E. coli* K12 W3110, △*galR,* △*galS,* △*pfkA*	This study
GR2PZ	*E. coli* K12 W3110, △*galR,* △*galS,* △*pfkA,* △*zwf*	This study
**Plasmids**		
pCW611	Ap^R^, λ‐Red recombinase under arabinose‐inducible *BAD* promoter, Cre‐recombinase under IPTG‐inducible *lacUV5* promoter, temperature sensitive origin	^36^
pMtrc9	*trc* promoter downstream of *lox66‐cat‐lox71* cassette	^37^

^a^CGSC, Coli Genetic Stock Center (New Haven, CT).

**Table 2:** Fermentation parameters obtained from the cultures of *E. coli* strains W3110, GR2, GR2P, and GR2PZ in R/2 medium containing 4 g/L glucose and 4 g/L galactose.

Strain	Maximum specific growth rate (μ_max_;/h)	Overall specific sugar consumption rate (g/gDCW/h)		Overall specific sugar consumption rate (mM/gDCW/h)		R_gal/glu_
		Glucose	Galactose	Glucose	Galactose	
W3110	0.5653 ± 0.01	1.3706 ± 0.04	0.0373 ± 0.00	7.614 ± 0.22	0.207 ± 0.00	0.03
GR2	0.4469 ± 0.02	1.3629 ± 0.12	0.1262 ± 0.08	7.572 ± 0.68	0.701 ± 0.44	0.09
GR2P	0.2993 ± 0.02	0.4714 ± 0.03	0.0552 ± 0.03	2.619 ± 0.16	0.307 ± 0.15	0.12
GR2PZ	0.2076 ± 0.01	0.4419 ± 0.01	0.0737 ± 0.02	2.455 ± 0.07	0.410 ± 0.13	0.17

### Galactose-to-glucose consumption ratio in *E. coli* GR2P, which lacked the galactose operon repressors and 6-phosphofructokinase, during glucose consumption

**Figure 3:**
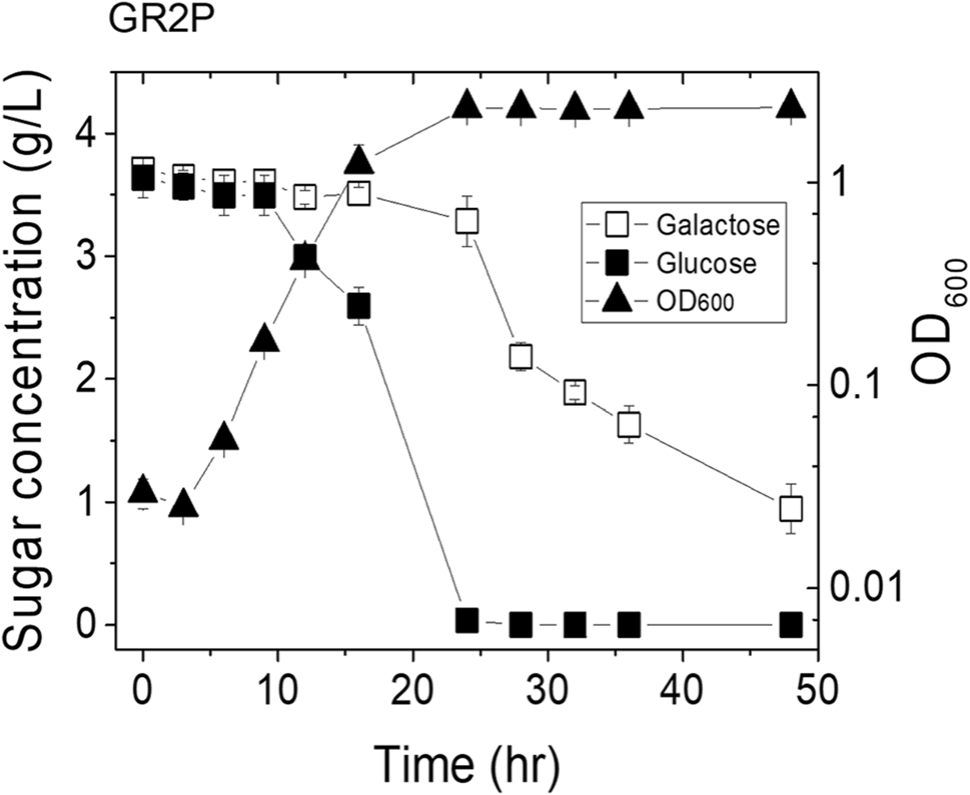
Time profiles of various parameters tested for *E. coli* strain GR2P grown in R/2 medium containing 4 g/L glucose and 4 g/L galactose.

Embden–Meyerhof–Parnas pathway (EMPP) was chosen as the next target to be engineered, because a metabolic intermediate glucose-6-phosphate is formed from both glucose and galactose. However, glucose-6-phosphate formation from both sugars are quietly differ from each other. To yield glucose-6-phosphate, glucose is catabolized by one-step reaction, while galactose is metabolized via Leior pathway^28^. Thus, it was expected that glucose and galactose consumption could be individually affected by modulation of EMPP. To further modulate R_gal/glu_, rather than overexpressing the galactose operon, we next knocked out the *pfkA* gene, which encodes ATP-dependent 6-phosphofructokinase, in *E. coli* strain GR2. As 6-phosphofructokinase is a key enzyme in the EMPP, we expected that the specific glucose consumption rate could be negatively affected by the lack of 6-phosphofructokinase in the resulting GR2P cells under the co-fermentation of glucose and galactose. In flask cultures, GR2P showed a maximum specific growth rate (µ_max_) of 0.2993/h with a short lag time, which was rather lower than the 0.5653/h and 0.4469/h obtained from cultures of W3110 and GR2, respectively (Fig. 3 and Table 2). This indicates that the additional knockout of the *pfkA* gene negatively affected cell growth. *E. coli* GR2P had completely consumed the glucose at 24 h of culture, yielding a specific glucose consumption rate of 0.4714 g/gDCW/h (Fig. 3 and Table 2). Meanwhile, the specific galactose consumption rate was 0.0552 g/gDCW/h, yielding an R_gal/glu_ of 0.12 (Table 2). Thus, our results indicate that the specific glucose consumption rate was negatively influenced by regulation of the EMPP in the engineered *E. coli*, whereas the specific galactose consumption rate was less sensitive to this manipulation.

### Galactose-to-glucose consumption ratio in *E. coli* GR2PZ, which lacked the galactose operon repressors, 6-phosphofructokinase, and glucose-6-phosphate dehydrogenase, during glucose consumption

**Figure 4:**
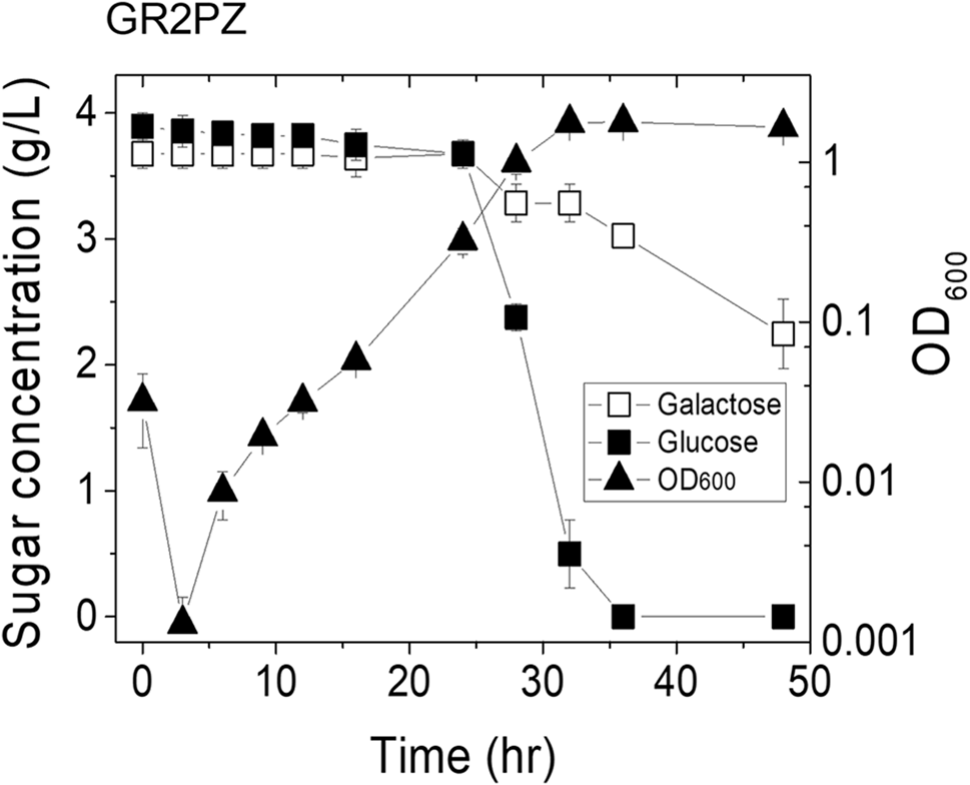
Time profiles of various parameters tested for *E. coli* strain GR2PZ grown in R/2 medium containing 4 g/L glucose and 4 g/L galactose.

A previous report found that *zwf*-mutant *E. coli*, which lacked glucose-6-phosphate dehydrogenase, showed carbon flow through the oxidative pentose phosphate pathway (OPPP)^29^. To additionally modulate the glucose consumption rate of our engineered strain, we knocked out the *zwf* gene in *E. coli* GR2P. As glucose-6-phosphate dehydrogenase is involved in converting glucose-6-phosphate to 6-phosphogluconate in the OPPP, we expected that the glucose consumption rate would be further reduced in the resulting GR2PZ strain, compared to the parent strain, GR2P. In the co-fermentation of glucose and galactose, GR2PZ showed a maximum specific growth rate of 0.2076 /h with a lag time, and thus had the lowest µ_max_ among the strains used in this study (Fig. 4 and Table 2). Furthermore, the sudden drop in cell mass was also observed at the very early stage (Fig. 4), which might be caused from the simultaneous knockout of the *pfkA* and *zwf* genes in *E. coli*. GR2PZ had completely consumed the glucose at 36 h of culture, yielding a specific glucose consumption rate of 0.4419 g/gDCW/h (Fig. 4 and Table 2). The specific galactose consumption rate was 0.0737 g/gDCW/h, for an R_gal/glu_ of 0.17.

### Galactose-to-glucose consumption ratio was further modulated by integration of a brief process optimization for initial sugar ratio

**Figure 5:**
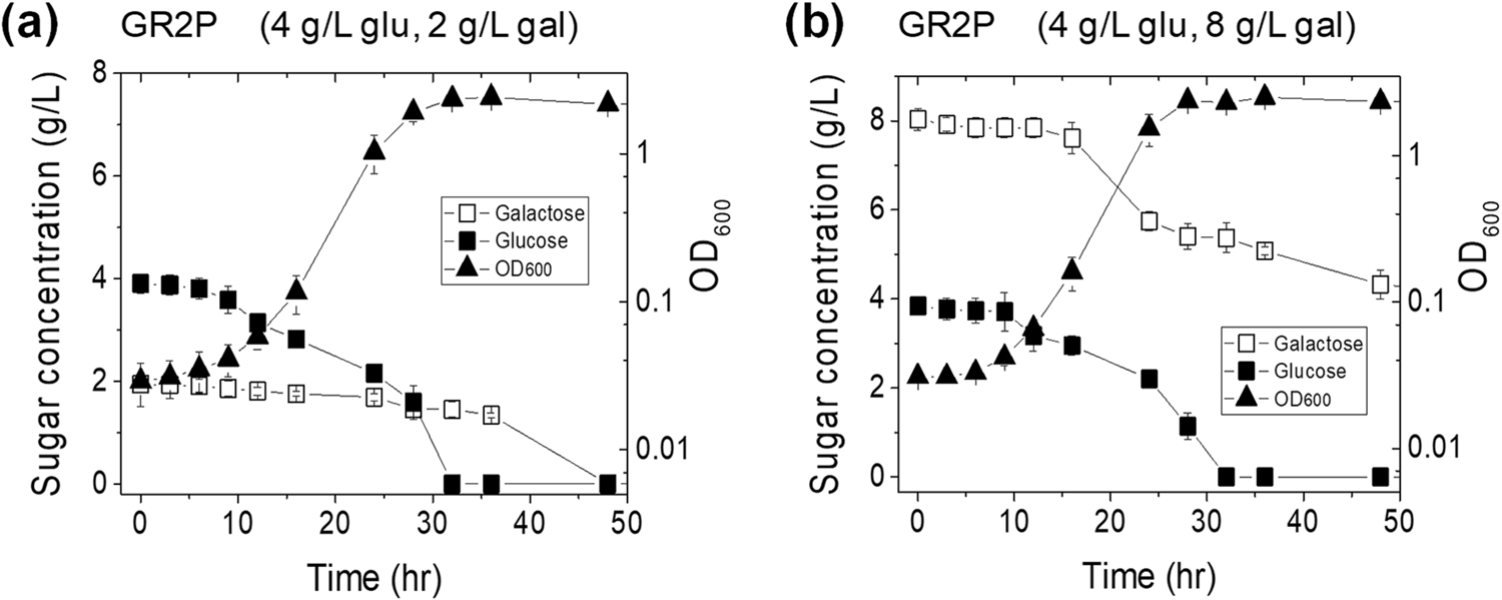
Time profiles of various parameters tested for *E. coli* strain GR2P grown in R/2 medium containing (**a**) 4 g/L glucose and 2 g/L galactose, and (**b**) 4 g/L glucose and 8 g/L galactose.

We demonstrated the control of glucose-to-galactose consumption ratio during the co-fermentation of glucose and galactose by engineering central sugar metabolism. Next, one of the engineered *E. coli* strains was applied to the process optimization for initial sugar ratio and concentrations, to see if the galactose-to-glucose consumption ratio could be further modulated by different initial sugar ratio and concentration. Among the engineered three strains, GR2P was randomly selected, and cultured in R/2 medium supplemented with sugars at the different initial ratio and concentration, such as 4 g/L glucose and 2 g/L galactose (4glu-2gal); and 4 g/L glucose and 8 g/L galactose (4glu-8gal) (Fig. 5). The culture parameters using GR2P in R/2 medium supplemented 4 g/L glucose and 4 g/L galactose (4glu-4gal) were obtained from Fig. 3. All parameters obtained from the culture conditions, such as 4glu-2gal, 4glu-4gal, and 4glu-8gal were listed in Table 3. *E. coli* GR2P achieved an R_gal/glu_ of 0.12, 0.12, and 0.67 under the condition of 4glu-2gal, 4glu-4gal, and 4glu-8gal, respectively (Table 3). Taken together, our results indicate that galactose-to-glucose consumption ratio was further modulated by integration of a brief process optimization together with the modulation of sugar metabolism.

**Table 3:** Fermentation parameters obtained from the cultures of *E. coli* strain GR2P in R/2 medium containing different initial glucose and galactose ratio and concentration.

Initial glucose and galactose (g/L, g/L)	Maximum specific growth rate (μ_max_;/h)	Overall specific sugar consumption rate (g/gDCW/h)		Overall specific sugar consumption rate (mM/gDCW/h)		R_gal/glu_
		Glucose	Galactose	Glucose	Galactose	
4, 2^a^	0.2733 ± 0.02	0.4469 ± 0.02	0.0539 ± 0.03	2.483 ± 0.13	0.299 ± 0.19	0.12
4, 4	0.2993 ± 0.02	0.4714 ± 0.03	0.0552 ± 0.03	2.619 ± 0.16	0.307 ± 0.15	0.12
4, 8^a^	0.2684 ± 0.03	0.4450 ± 0.03	0.2961 ± 0.06	2.472 ± 0.19	1.306 ± 0.31	0.67

^a^Overall specific sugar consumption rate was determined after normalization of 6-h lag.

## Discussion

Galactose produced by a marine biomass is a promising fermentable sugar for the production of biofuels and biochemicals. A recent report described an engineered *E. coli* strain capable of consuming galactose at the same rate as glucose^20^. In the same study, an R_gal/glu_ of nearly 1.0 was achieved by overexpressing the galactose operon in a mutant *E. coli* lacking the galactose operon repressor^20^. However, the fine-tuned control of galactose consumption by *E. coli* has not previously been reported in a co-fermentation with glucose. In this study, we demonstrate that the galactose consumption rate may be finely controlled during co-fermentation with glucose by comparing our engineered *E. coli* strains and their parent W3110. Strains W3110, GR2, GR2P, and GR2PZ achieved R_gal/glu_ values of 0.03, 0.09, 0.12, and 0.17, respectively, during the co-fermentation of these sugars. Meanwhile, R_gal/glu_ of 0.67 was achieved by a brief process optimization using GR2P strain.

We found that the galactose-to-glucose consumption ratio could be slightly modulated by disrupting the galactose operon repressors encoded by the *galRS* genes, and further modulated by regulating the central metabolic pathway. The *galRS*-mutant *E. coli* strain, GR2, had an R_gal/glu_ ratio of 0.09, compared to the 0.03 ratio of parent strain W3110. Products of the *galRS* genes have been reported to negatively regulate galactose metabolism by binding upstream of the galactose operon^19,30–32^. Thus, the inactivation of galactose operon repressors could logically increase the specific galactose consumption rate in *E. coli* while leaving the specific glucose consumption rate unchanged. Indeed, in the co-fermentation of glucose and galactose, GR2 and W3110 showed specific galactose consumption rates of 0.1262 g/gDCW/h and 0.0373 g/gDCW/h, respectively, while yielding specific glucose consumption rates of 1.3629 g/gDCW/h and 1.3706 g/gDCW/h (Fig. 2, Table 2).

In the co-fermentation of glucose and galactose using strains GR2P and GR2PZ, in which the central metabolic pathway was further modified by knockout of the *pfkA* and *pfkA-zwf* genes, respectively, we obtained R_gal/glu_ ratios of 0.12 and 0.17, respectively. For GR2P and GR2PZ, the specific consumption rates for both sugars were decreased compared to that of parent strain GR2. Glucose consumption was more sensitive than galactose consumption to modulation of the central metabolic pathway: The specific glucose consumption rate decreased from 1.3629 g/gDCW/h (GR2) to 0.4714 g/gDCW/h (GR2P; 65% decrease) and 0.4419 g/gDCW/h (GR2PZ; 68% decrease), while the specific galactose consumption rate decreased from 0.1262 g/gDCW/h (GR2) to 0.0552 g/gDCW/h (GR2P; 56%) and 0.0737 g/gDCW/h (GR2PZ; 41%). R_gal/glu_ ratios of 0.12 and 0.17 were obtained for the co-fermentation of glucose and galactose by strains GR2P and GR2PZ, respectively (Fig. 4, Table 2). We do not yet know why glucose consumption was more sensitive than galactose consumption to the *pfkA* and *pfkA-zwf* mutations. However, this may relate to a previous report that deletion of the *zwf* gene altered the metabolism of the mutant cells by changing the redox balance^33–35^.

The engineered GR2P and GR2PZ strains utilized both sugars simultaneously in the presence of the major CCR. Under the *galRS* double-knockout condition, it seems that the repression of galactose metabolism by the CCR was slightly leaky, which was supported by Table 2 and Fig. 2. The *galRS* were knocked-out in GR2P and GR2PZ strains also, which means that two strains were leaky in CCR, too. Thus, we believe that the simultaneous utilization of both sugars was not resulted from the complete deregulation of CCR. However, the simultaneous utilization of both sugars in GR2P and GR2PZ strains could be explained by dramatic decrease of specific glucose consumption rate compared to the parent GR2 strain (Table 2), which was more sensitive compared to the decrease of specific galactose consumption.

In conclusion, we herein report the regulation of the galactose-to-glucose consumption ratio by engineering *E. coli* strains for the co-fermentation of these sugars. We achieved R_gal/glu_ values of 0.03, 0.09, 0.12, and 0.17 in the co-fermentation of glucose and galactose using *E. coli* strains W3110, GR2, GR2P, and GR2PZ, respectively. The R_gal/glu_ value of 0.67 was achieved by a brief process optimization for initial sugar ratio using GR2P strain. In the engineered *E. coli* strains, the galactose-to-glucose consumption ratio could be changed by activation of the galactose operon and modulation of central metabolic pathways (EMPP and OPPP) in the co-fermentation of both sugars. The strategy reported in this study can help regulate the galactose-to-glucose consumption ratio in a microbial co-fermentation of both sugars from galactose-rich biomass. Furthermore, the repression of carbohydrate metabolism might be applicable to enhance acetyl-CoA pool from another carbon source which is uncommon to *E. coli*, such as polyethylene.

## Materials and methods

### Microbial strains and plasmids

Details on the utilized engineered *E. coli* strains and their parent strain, K-12 W3110, are listed in Table 1. Plasmid pCW611, which harbored the genes encoding lambda red recombinase and Cre-recombinase, was used for gene knockout in *E. coli*^36^. Plasmid pMtrc9, which contained the *lox66-cat-lox77* cassette, was used as a PCR template to amplify the DNA fragment for target gene knockout^37^. Details on the utilized plasmids are presented in Table 1.

### Media and culture conditions

*Escherichia coli* cells were cultured in R/2 medium supplemented with the indicated carbon sources. R/2 medium (pH 6.8) contained 2 g (NH_4_)_2_HPO_4_, 6.75 g KH_2_PO_4_, 0.7 g MgSO_4_·7H_2_O, 0.85 g citric acid, and 5 mL trace metal solution per liter^38^. The trace metal solution contained (per liter): 0.1 M HCl, 10 g FeSO_4_·7H_2_O, 2.25 g ZnSO_4_·7H_2_O, 0.58 g MnSO_4_·5H_2_O, 1 g CuSO_4_·5H_2_O, 0.1 g (NH_4_)_6_Mo_7_O_24_·4H_2_O, 0.02 g Na_2_B_4_O_7_·10H_2_O, and 2 g CaCl_2_·2H_2_O^38^.

To prepare the seed culture, a colony grown on an LB agar plate was inoculated into 25-mL test tubes containing 5 mL R/2 medium. The test tubes were incubated in a shaking incubator (IST-4075, JEIOTECH, Korea) at 37 °C and 200 rpm. The next day, 2.2 mL of seed culture was transferred to each 500-mL flask containing 220 mL R/2 medium. For co-fermentation experiments, 3 g/L glucose and 3 g/L galactose were supplemented in the medium as carbon sources.

### Construction of knockout mutants

Wild type *E. coli* strain K-12 W3110 was used for the construction of all studied mutants. The quadruple gene knockout mutant, GR2PZ (described here as an example) was constructed using the modified one-step inactivation method^36^ to knock out the *galR*, *galS*, *pfkA*, and *zwf* genes. The primers listed in Table S1 were designed from the sequences of the target genes and plasmid pMtrc9, which contained the *lox66*-*cat*-*lox71* cassette. To construct a linear DNA fragment for knockout of the *galR*, *galS*, *pfkA* and *zwf* genes, we used primer pairs galR-KO-F1 and galR-KO-R1, galS-KO-F1 and galS-KO-R1, pfkA-KO-F1 and pfkA-KO-R1, and zwf-KO-F1 and zwf-KO-R1, respectively. The resulting DNA fragments were individually PCR amplified using the corresponding primer pairs: galR-KO-F2 and galR-KO-R2, galS-KO-F2 and galS-KO-R2, pfkA-KO-F1 and pfkA-KO-R1, and zwf-KO-F2 and zwf-KO-R2, respectively. The final fragments were individually electroporated into *E. coli* cells harboring the pCW611 helper plasmid, using a BTX ECM 630 electroporator (BTX, San Diego, CA, USA; 1.8 kV, 25 μF, 200Ω, 1-mm electroporation cuvettes). Using pCW611, which contains both lambda red recombinase and the Cre recombinase, target gene knockout in *E. coli* was conducted as previously described^36^. Mutation of the target genes, *galR, galS, pfkA,* and *zwf*, was validated by colony PCR using primer pairs galR-KO-F1 and galR-KO-R1, galS-KO-F1 and galS-KO-R1, pfkA-KO-F1 and pfkA-KO-R1, and zwf-KO-F1 and zwgal-KO-R1, respectively. Finally, plasmid pCW611 was removed from the mutant *E. coli* cells by incubation at 42℃. Double and triple gene knockout mutants, GR2 and GR2P were also constructed by knockout combination of the corresponding genes in the same manner.

### Determination of the galactose-to-glucose consumption ratio

To determine the galactose-to-glucose consumption ratio during glucose consumption, *E. coli* cells were cultured in 500-mL flasks containing R/2 medium supplemented with 4 g/L glucose and 4 g/L galactose, at 37 °C and 200 rpm. One milliliter of culture broth was taken at 0, 3, 6, 9, 12, 16, 24, 28, 32, 36, and 48 h and used to determine the residual glucose and galactose concentrations (see below for analytical methods). Another 1 mL of broth was taken at the same time and used to determine the dry cell weight. From these analyses, the specific glucose consumption rate (R_glu_) was determined, while the specific galactose consumption rate (R_gal_) was determined during glucose consumption. The galactose-to-glucose consumption ratio, R_gal/glu_, was determined. All flask fermentations were independently performed in triplicate.

### Analytic methods

Cell growth was monitored by determining the absorbance at 600 nm using a spectrophotometer (HIACHI, JP/U-1900, Tokyo, Japan). For determination of the glucose and galactose concentrations, culture broth supernatants were prepared by centrifugation followed by filtration using a 0.22-μm pore syringe filter. Samples were applied to high performance liquid chromatography (HPLC; 1100 series, Agilent Technologies, Santa Clara, CA, USA) equipped with a refractive index detector (RID; RI-101, Shodex, Klokkerfaldet, Denmark)^39–42^. A MetaCarb 87H column (Agilent Technologies) was used with 0.01 N H_2_SO_4_ as a mobile phase at 25 °C, with a flow rate of 0.5 mL/min.

## Supplementary information

Supplementary Table.
