# Supplementary material for: Control of the galactose-to-glucose consumption ratio in co-fermentation using engineered Escherichia coli strains
Source: Sci Rep. 2020 Jul 22;10:12132. doi: 10.1038/s41598-020-69143-3 (PMC7376015; doi:10.1038/s41598-020-69143-3)
Supplement: Supplementary file 1 — Supplementary Table. [file 41598_2020_69143_MOESM1_ESM.docx]

**Supplementary material**

**Control of the galactose-to-glucose consumption ratio in co-fermentation using engineered *Escherichia coli* strains**

Hyeon Jeong Seong, Ji Eun Woo & Yu-Sin Jang^*^

Department of Agricultural Chemistry and Food Science Technology, Division of Applied Life Science (BK21 Plus Program), Institute of Agriculture & Life Science (IALS), Gyeongsang National University, Jinju 52828, Republic of Korea

*Corresponding author.

E-mail address: jangys@gnu.ac.kr (Y.-S. Jang)

**Table S1** Oligonucleotides used in this study ^1^

| Name | Sequences (5’ → 3’) |
| --- | --- |
| **Oligonucleotides** |  |
| galR-KO-F1 | ATGTAAGCGTTTACCCACTAAGGTATTTTCGACACTATAGAACGCGGCCG |
| galR-KO-R1 | TTACTGGCGCTGGAATTGCTTTAACTGCGGCCGCATAGGCCACTAGTGGA |
| galR-KO-F2 | AAAACACGCCACCCCTTGAACCAACGGGCGTTTTCCGTAACACTGAAAGAATGTAAGCGTTTACCCACTA |
| galR-KO-R2 | TTGATAATGGTCAGGCGCGGTTGATTCGCCGTCGCCAGACCATCGAAGAATTACTGGCGCTGGAATTGCT |
| galS-KO-F1 | ACTGTGAAATCACTCACAGATTGAAAGCGGGACACTATAGAACGCGGCCG |
| galS-KO-R1 | CAGTCATTTACTGCAATCTCATAACAGGTACCGCATAGGCCACTAGTGGA |
| galS-KO-F2 | CGGGTTACAACGTTAAAACGGTGCAATCATAGCTATCACATTGTTAAGATACTGTGAAATCACTCACAGA |
| galS-KO-R2 | TCGATTCACGAAGTCCTGTATTCAGTGCTGACAAAATAGCCGCCAGCAAGCAGTCATTTACTGCAATCTC |
| zwf-KO-F1 | TAAAATAACCATAAAGGATAAGCGCAGATAGACACTATAGAACGCGGCCG |
| zwf-KO-R1 | TACCGGGTTAGTTAACTTAAGGAGAATGACCCGCATAGGCCACTAGTGGA |
| zwf-KO-F2 | CCTGAAAGTGTAAAAATTGTTCTACAATCTGCGCAAGATCATGTTACCGGTAAAATAACCATAAAGGATA |
| zwf-KO-R2 | AAAGCAGTACAGTGCACCGTAAGAAAATTACAAGTATACCCTGGCTTAAGTACCGGGTTAGTTAACTTAA |
| pfkA-KO-F1 | GGTAAAGGAATCTGCCTTTTTCCGAAATCAGACACTATAGAACGCGGCCG |
| pfkA-KO-R1 | TACTATTTGCACATTCGTTGGATCACTTCGCCGCATAGGCCACTAGTGGA |
| pfkA-KO-F2 | CCTGATAAGCGAAGCGCATCAGGCATTTTTGCTTCTGTCATCGGTTTCAGGGTAAAGGAATCTGCCTTTT |
| pfkA-KO-R2 | ATACCGCCATTTGGCCTGACCTGAATCAATTCAGCAGGAAGTGATTGTTATACTATTTGCACATTCGTTG |

**Reference**

1 Woo, J. E., Seong, H. J., Lee, S. Y. & Jang, Y.-S. Metabolic engineering of *Escherichia coli* for the production of hyaluronic acid from glucose and galactose. *Front. Bioeng. Biotechnol.* **7**, 351 (2019).
